# Coordinated international action to accelerate genome-to-phenome with FAANG, the Functional Annotation of Animal Genomes project

**DOI:** 10.1186/s13059-015-0622-4

**Published:** 2015-03-25

**Authors:** The FAANG Consortium, Leif Andersson, Alan L Archibald, Cynthia D Bottema, Rudiger Brauning, Shane C Burgess, Dave W Burt, Eduardo Casas, Hans H Cheng, Laura Clarke, Christine Couldrey, Brian P Dalrymple, Christine G Elsik, Sylvain Foissac, Elisabetta Giuffra, Martien A Groenen, Ben J Hayes, LuSheng S Huang, Hassan Khatib, James W Kijas, Heebal Kim, Joan K Lunney, Fiona M McCarthy, John C McEwan, Stephen Moore, Bindu Nanduri, Cedric Notredame, Yniv Palti, Graham S Plastow, James M Reecy, Gary A Rohrer, Elena Sarropoulou, Carl J Schmidt, Jeffrey Silverstein, Ross L Tellam, Michele Tixier-Boichard, Gwenola Tosser-Klopp, Christopher K Tuggle, Johanna Vilkki, Stephen N White, Shuhong Zhao, Huaijun Zhou

**Affiliations:** Science for Life Laboratory Uppsala, Department of Medical Biochemistry and Microbiology, Uppsala University, Uppsala, SE 751 23 Sweden; Department of Animal Breeding and Genetics, Swedish University of Agricultural Sciences, Uppsala, SE-750 07 Sweden; The Roslin Institute and Royal (Dick) School of Veterinary Studies, University of Edinburgh, Edinburgh, EH25 9RG UK; School of Animal and Veterinary Sciences, The University of Adelaide, Roseworthy, SA 5371 Australia; Invermay Agricultural Centre, AgResearch Limited, Mosgiel, 9053 New Zealand; College of Agriculture and Life Sciences, University of Arizona, Tucson, AZ 85719 USA; National Animal Disease Center, United States Department of Agriculture, Agricultural Research Service, Ames, IA 50010 USA; Avian Disease and Oncology Laboratory, United States Department of Agriculture, Agricultural Research Service, East Lansing, MI 48823 USA; European Molecular Biology Laboratory European Bioinformatics Institute, Hinxton, Cambridge, CB10 1SD UK; Livestock Improvement Corporation, Hamilton, 3284 New Zealand; Agriculture Flagship, Commonwealth Scientific and Industrial Research Organisation, St Lucia, 4067 Brisbane Australia; Division of Animal Sciences, University of Missouri, Columbia, MO 65211 USA; UMR1388 Génétique, Physiologie et Systèmes d’Elevage (GenPhySE), French National Institute for Agricultural Research (INRA), F-31326 Castanet-Tolosan, France; UMR1313 Génétique Animale et Biologie Intégrative (GABI), French National Institute for Agricultural Research (INRA), F-78352 Jouy-en-Josas, France; Animal Breeding and Genomics Centre, Wageningen University, 6708 PB Wageningen, The Netherlands; Biosciences Research Division, Department of Environment and Primary Industries Victoria, Bundoora, 3083 Australia; Dairy Futures Cooperative Research Centre, Bundoora, 3083 VIC Australia; La Trobe University, Bundoora, 3086 VIC Australia; Key Laboratory for Animal Biotechnology of Jiangxi Province and the Ministry of Agriculture of China, Jiangxi Agricultural University, Jiangxi, 330029 People’s Republic of China; Department of Animal Sciences, University of Wisconsin-Madison, Madison, WI 53706 USA; Department of Agricultural Biotechnology, Seoul National University, Seoul, Republic of Korea; Animal Parasitic Diseases Laboratory, United States Department of Agriculture, Agricultural Research Service, Beltsville, MD 20705 USA; School of Animal and Comparative Biomedical Sciences, University of Arizona, Tucson, AZ 85721 USA; Animal Productivity Group, AgResearch Limited, Mosgiel, 9053 New Zealand; Centre for Animal Science, Queensland Alliance for Agriculture & Food Innovation, University of Queensland, St Lucia, QLD 4067 Australia; Department of Basic Sciences, College of Veterinary Medicine and Institute for Genomics, Biocomputing and Biotechnology, Mississippi State University, Mississippi, MS 39762 USA; Comparative Bioinformatics, Centre for Genomic Regulation (CRG), Dr. Aiguader 88, 08003 Barcelona, Spain; National Center for Cool and Cold Water Aquaculture, United States Department of Agriculture, Agricultural Research Service, Kearneysville, WV 25430 USA; Livestock Gentec Centre, Department of Agricultural, Food and Nutritional Science, University of Alberta, Edmonton, Alberta T6G 2C8 Canada; Department of Animal Science, Iowa State University, Ames, IA 50011 USA; US Meat Animal Research Center, United States Department of Agriculture, Agricultural Research Service, Clay Center, NE 68933 USA; Institute of Marine Biology, Biotechnology and Aquaculture, Hellenic Centre for Marine Research, 71003 Heraklion, Greece; Department of Animal and Food Sciences, University of Delaware, Newark, DE 19716 USA; Animal Production and Protection, United States Department of Agriculture, Agricultural Research Service Aquaculture, Beltsville, MD 20705 USA; CSIRO Agriculture, Commonwealth Scientific and Industrial Research Organisation, St Lucia, 4067 Australia; Green Technology, Natural Resources Institute Finland, 31600 Jokioinen, Finland; Animal Disease Research Unit, United States Department of Agriculture, Agricultural Research Service, Pullman, WA 99164-6630 USA; Department of Veterinary Microbiology and Pathology, Washington State University, Pullman, WA 99164-7040 USA; Key Laboratory of Agricultural Animal Genetics, Breeding and Reproduction of Ministry of Education of China, Huazhong Agricultural University, Wuhan, Hubei 430070 People’s Republic of China; Department of Animal Science, University of California, Davis, CA 95616 USA

## Abstract

We describe the organization of a nascent international effort, the Functional Annotation of Animal Genomes (FAANG) project, whose aim is to produce comprehensive maps of functional elements in the genomes of domesticated animal species.

**Electronic supplementary material:**

The online version of this article (doi:10.1186/s13059-015-0622-4) contains supplementary material, which is available to authorized users.

## Predictive biology: from sequence to consequence

Most phenotypes are complex and quantitative in nature, and a major goal of biological research lies in using genome information to predict such complex outcomes, whether it is the efficacy of a drug, susceptibility to cancer, or the performance of the daughters of an elite dairy bull. Many of the recent advances in biology have been driven by genome sequence information. The capability to sequence and decipher the instructions encoded in complex animal genomes quickly and at modest cost is now well established. The next challenge is to be able to read the subtlety and complexity of these instructions and to predict the resulting phenotypes, that is, to predict the consequences encoded in sequences. While significant progress in functional genome annotation has been made using various human cell types [1], we argue that filling the genotype-to-phenotype gap requires functional genome annotation of species with substantial phenotype information.

## The unique value of domesticated animal species for accelerating our understanding of genomes and phenomes

Research on domesticated animals has important scientific and socioeconomic impacts, including contributing to medical research, improving the health and welfare of companion animals, and underpinning improvements in the animal sector of agriculture. A key to these impacts is the wealth of genetic and phenotypic diversity among domesticated animals, coupled with research to elucidate the genetic architecture underlying quantitative traits.

### From association to causation: pioneering success in domesticated species

Deep pedigrees with extensive phenotypic records, genetic and phenotypic diversity shaped by natural and artificial selection, and the latest molecular genomics and statistical tools provide an opportunity to understand the relationship between genotype and phenotype in outbred domesticated and farmed animal species [2]. We cite four examples of past successes. First, the identification of a single base-pair change as the causal genetic variant for the complex callipyge muscle hypertrophy phenotype in sheep [3]. Second, the finding that a single nucleotide change in the 3’-untranslated region of the sheep myostatin gene creates a new microRNA binding site that decreases myostatin protein expression [4]. Third, the identification of a single nucleotide change in an *IGF2* intron that is the causal mutation for a quantitative trait locus with effects on muscle growth and fat depth in pigs [5]. Finally, the finding that a premature stop codon in the *DMRT3* gene has a major effect on the pattern of locomotion in horses [6]. Much of the genetic variation underlying quantitative traits is likely to be located in regulatory sequences [7], and two of the examples cited above [3,5] demonstrate the importance of epigenetic mechanisms in determining complex phenotypes.

### Evolution, selection, adaptation

The study of genomes of domesticated animals provides insight into evolution, adaptation and genetic selection. Domesticated and farmed animals represent a wide evolutionary spectrum from bees, through shellfish, fish, birds and mammals, and analyses of their genomes have revealed relationships between sequence and function [8-12]. Genome-wide analysis of domesticated species and their putative wild ancestors has shed light on domestication [8,13-15]. Importantly, the footprint of artificial selection can also be detected and provides glimpses of the relationship between sequence and selected phenotypes [16-18].

### Biomedical models

Several domesticated animal species are widely used to model human biology, including the pig, sheep, chicken and dog. However, while coding sequence variants can be major determinants of phenotype as exemplified by many monogenic inherited diseases, attempts to recapitulate the disease phenotype in genetically modified mice often fail [19]. This lack of accurate translation to human biology demonstrates the need for a better understanding of the genotype-to-phenotype relationship [20], potentially through the use of additional species that better approximate human physiology [21].

### Modeling animals as systems: success in phenotypic selection but little mechanistic knowledge

Animals are complex systems in which predicting phenotype from genotype (sequence) is challenging. However, quantitative geneticists and animal breeders have been remarkably successful at developing statistical animal models that are effective predictors of future performance [22]. The accuracy of these models has been increased by using high-density single nucleotide polymorphism genotypes [22,23]. Further improvements can be achieved through the use of genome sequence data [24-26] and by adding knowledge of the likely effects of the sequence variants, whether coding or regulatory [27]. However, while artificial selection acting on the enormous underlying genetic diversity has made improvements in traits of economic importance, there is little understanding of the biological mechanisms underpinning such phenotypes.

## Recent progress in animal genome sequencing provides new opportunities in elucidating the genotype-to-phenotype connection

Coordinated genome-wide identification of functional elements in multiple species would be an invaluable resource for the dissection of genotype-to-phenotype relationships. The evolutionary breadth of the Encyclopedia of DNA Elements (ENCODE) projects has been expanded from humans to classical model species (mouse [28,29], *Drosophila* [30], *Caenorhabditis elegans* [31] and zebrafish [32]). However, transcriptome complexity differs significantly between species [33]; in general, extrapolation of regulatory sequence data across species has not proven useful [34]. In line with previous evidence, the mouse ENCODE project provided multiple lines of evidence that gene expression and its underlying regulatory programs have substantially diverged between the human and mouse lineages, although a subset of core regulatory programs is largely conserved [29]. Thus, additional sampling of species, especially those with deep phenotypic records, is needed to fully understand how these functional elements define the timing, amplitude and response to developmental and environmental cues [35].

A prerequisite for mapping functional elements is a reference genome assembly. Reference genome sequences have been established for a range of important domesticated animals (Additional file 1). However, the annotation of these genome sequences is currently limited to gene models deduced using RNA expression and DNA variation data. Thus, in comparison to human and mouse, the complexity of the transcriptomes in domesticated animals is inadequately characterized. This is exacerbated by the fact that while 70% to 90% of the coding elements can be readily identified, there is little information on noncoding genes, and even less on the regulatory sequences that often underlie complex traits.

The ENCODE and epigenome consortia have already demonstrated that improved functional annotation is most efficiently delivered collaboratively [1,28-32,36]. Thus, in combination with filling the gap in deriving phenotype from genotype described above, this advantage is a strong motivation for an internationally coordinated Functional Annotation of Animal Genomes (FAANG) project as proposed below.

## The FAANG Consortium

In January 2014, a workshop was convened by the Animal Biotechnology Working Group of the EU-US Biotechnology Research Task Force in San Diego, CA, USA. During this workshop, and in subsequent discussions, basic principles were laid out to establish the FAANG Consortium and to outline plans for a FAANG project (see below). The aim of the Consortium is to produce comprehensive maps of functional elements in the genomes of domesticated animal species based on common standardized protocols and procedures. The FAANG Consortium signatories are committing to work within the FAANG community to define and improve experimental, metadata and bioinformatics standards; ensure that experiments conducted to produce functional annotation adhere to these standards; and release all the experimental and metadata in an open access manner, rapidly and before publication, in accordance with the Toronto Statement [37].

A web portal has been established to consolidate and distribute information on the FAANG Consortium (standardized protocols and pipelines of analysis, data summaries, and publications) and as a means for new participants to join the Consortium [38]. Additional details on the FAANG Consortium, including current membership and goals, can also be found on the web portal.

## Delivering the FAANG project

The human ENCODE project cost over $150 million and involved at least 442 scientists in 32 institutions around the world. Lessons learned from this project and advances in high-throughput technologies have transformed the ease and efficiency with which this type of project can be executed. A coordinated effort to generate data from similar tissues using common core assays to minimize redundancy and leverage existing activity will enable the FAANG project to make significant progress in a cost-effective manner. ENCODE-type data will be generated at a fraction of the original cost and in a distributed way, thanks to the modular nature of experiments.

### Parallel sample and data collection from species ready to implement FAANG

A high-quality reference genome assembly is a prerequisite to initiate a functional annotation effort. Consequently, we propose to start by selecting taxonomically diverse species with high-quality genome assemblies. These species need to have the support of their research community and a critical mass of investigators, as demonstrated by expression of interest and willingness to use core assays and a common data-sharing infrastructure. Currently, domesticated animal species that meet this requirement include chicken, pig, cattle and sheep. We note, however, that research on other species (for example, goat, salmon and catfish) is rapidly expanding the range of genomes suited for a FAANG approach (Additional file 1).

The first phase of the FAANG project will focus on sampling biological replicates representing a limited number of specific biological states to maximize comparisons across species. Where possible, animals with minimal genetic diversity within a species will be sampled. For example, highly inbred lines of chicken can be used. While each species’ community will decide on a particular breed, genetic line or cross, FAANG members are committed to collecting, storing and sharing tissues for initial data collection as well as holding them in reserve for future additional assays. Similarly to recent phases of ENCODE and modENCODE [29,39], FAANG will mostly focus on tissue samples. A first core set of tissues directly related to the large number of quantitative phenotypes available in several domesticated species has been defined. This includes skeletal muscle, adipose, liver, and tissues collected from the reproductive, immune and nervous systems. We believe this will allow a more direct connection between genome function and quantitative phenotype than the transformed cell lines used extensively in the first phase of the ENCODE project [39]. Both male and female progeny will be sampled at neonatal and mature stages.

### FAANG data types

Both ENCODE and the International Human Epigenome Consortium have defined robust experimental protocols [40]. We will use these standards as a baseline, adapting them where necessary to reflect the complexities of animal breeds and the different tissues available for animal-based experiments. We plan to employ a few specific core assays, which for the most part employ technologies that work across all targeted species (RNA sequencing, chromatin accessibility, and histone marks) as well as have selected laboratories run these assays for the community with standard protocols (Box 1). Additional assays may be performed by individual research groups based upon specific needs and research interests.

### Common data infrastructure

Effective coordination, data management and robust quality control (QC) are essential to converting data generated across multiple laboratories into knowledge. The FAANG consortium will promote standardization of experimental protocols and procedures in computational analysis. A sampling coordination task force will promote standards for sampling and storing conditions, including the documentation of animal origin and environmental conditions. A FAANG Data Coordination Centre (DCC) and a Data Analysis Centre (DAC) will be established to ensure high-quality and standardized data generation and analysis, and accessibility of the data to the wider community [41]. The FAANG DCC will work with the Sequence, Variation and Sample archives at European Molecular Biology Laboratory European Bioinformatics Institute and the National Center for Biotechnology Information to ensure the data are deposited, with suitable metadata descriptions, in the appropriate archives. In addition, the FAANG DCC will provide quality-controlled data to resources like Ensembl, so that the improved annotation is available to the broadest audience possible. Appropriate metadata and data quality standards for test samples will be defined, and the DCC will help to collect and QC data generated by FAANG partners. The DCC will help groups to appropriately archive sample data and metadata and provide mechanisms to share and access data [37]. Key tasks such as mapping the primary sequence data to the appropriate reference genome will be performed by the DCC. The FAANG DAC will consist of distributed groups to establish the best bioinformatic pipelines to analyze FAANG consortium data, and will work closely with the DCC to ensure appropriate QC standards are defined.

### Future expansion of covered species and diversity within and between species

As reference genomes for new species are added across the tree of life, new insights can be obtained through functional analysis of such species. Thus, it will be important to continue to expand the evolutionary diversity of FAANG over time.

It is expected that additional insights will be gained by expanding the genetic diversity within a given species. This fine-scale detail will provide invaluable insight into genetic regulation of phenotypic diversity at a mechanistic level. Furthermore, additional samples and species relevant to specific groups will be collected. New samples may include rumen tissues from ruminant species, mammary tissue from mammals and fiber-producing tissue in animals raised for fiber production. Many aquatic species are able to produce interesting atypical progeny (double haploid and sex-reversed progeny) and both poultry and aquatic species produce very large full-sibling cohorts.

## Impact of FAANG

Similar to the ENCODE projects, the FAANG functional maps will generate a comprehensive data resource to be used by multiple groups, over a long time, for multiple purposes [42]. Thanks to this organized effort in coordination and standardization, individual research groups will be able to effectively use - and refer to - FAANG datasets, as well as contribute their own datasets from specific genome-to-phenome investigations in different species.

Overall, we predict completing the aims of the FAANG project will enable the application of molecular phenotypes to the prediction of complex phenotypes and further our understanding of additive and non-additive genetic mechanisms such as dominance and epistasis. Such knowledge can be applied to animal production, human and animal health, evolution, adaptation, and understanding the role of animals in their ecosystem. There is also evidence that early developmental influences can affect transiently inherited acquired traits, indicating that epigenetic modifications to the genome may be another important factor in understanding the inheritance of complex traits. FAANG will provide critical basic information, which will be used to improve food production and inform studies of agriculture, biomedical science, evolution and the environment.

## Box 1

**Core assays:**

**Transcribed loci** - Identifying the transcribed elements of the genome is a key starting point for functional annotation. RNA-sequencing data generated from libraries prepared using stranded protocols [43], the species of interest, and a wide range of tissues, cells and states are critical to describe transcript complexity, including alternative splicing and non-coding RNAs [44-46].

**Chromatin accessibility and architecture** - Assay for transposase-accessible chromatin sequencing (ATAC-seq), which is based on direct *in vitro* transposition of sequencing adaptors into native chromatin, represents a rapid and sensitive alternative to DNaseI footprinting [47] for detecting open chromatin [48,49]. Chromatin immunoprecipitation sequencing (ChIP-seq) to identify sites bound by the highly conserved insulator-binding protein CCCTC-binding factor can be useful for bridging the gap between gene expression and nuclear organization [50].

**Histone modification marks** - The presence of modified histones and the characterization of the sequences to which they are bound will be assayed by well-standardized ChIP-seq assays using validated antibodies that work across a broad range of species. We will start with four histone modification marks among those found most informative by the ENCODE projects [1,51]:

- Histone H3 lysine 4 trimethylation (H3K4me3), which correlates with promoters of active genes and transcription start sites;
- Histone H3 lysine 27 trimethylation (H3K27me3), which marks genes that have been silenced through regional modification;
- Histone H3 lysine 27 acetylation (H3K27ac), which marks active regulatory elements, and may distinguish active enhancers and promoters from their inactive counterparts;
- H3 lysine 4 monomethylation (H3K4me1), which marks regulatory elements associated with enhancers and other distal elements, but is also enriched downstream of transcription start sites.

***Additional assays:***

**Methylation** - DNA methylation is a major epigenetic mark and a well-known regulator of gene expression. Genome-wide analysis of 5-methylcytosines can be performed at nucleotide-level resolution by whole-genome or reduced representation bisulfite sequencing [52].

**Transcription factor binding sites** - ChIP-seq assays can be used to identify sequences bound by specific proteins [40]. However, generating and validating the antibodies necessary will be the main challenge in mapping binding sites for the diversity of transcription factors in species of interest.

**Genome conformation** - Methods of chromosome conformation capture allow the study of the genome-wide chromatin interactome. Hi-C is an upgraded method that provides information about distal elements that, while far apart in the primary sequence, are brought together because of chromosome folding [53,54]. Hi-C results can be optimally integrated with those from other assays of chromatin accessibility (for example, ATAC-seq).

## Additional file

Additional file 1: Table S1. Reference species and publications of livestock genomes.
