# Supplementary material for: Coordinated international action to accelerate genome-to-phenome with FAANG, the Functional Annotation of Animal Genomes project
Source: Genome Biol. 2015 Mar 25;16(1):57. doi: 10.1186/s13059-015-0622-4 (PMC4373242; doi:10.1186/s13059-015-0622-4)
Supplement: Additional file 1: Table S1. — Reference species and publications of livestock genomes. [file 13059_2015_622_MOESM1_ESM.docx]

| Species | Year | Reference |
| --- | --- | --- |
| fugu (*Fugu rubripes*) | 2002 | Aparicio et al. 2002. Whole-Genome Shotgun Assembly and Analysis of the Genome of Fugu rubripes. Science 297: 1301-1310 |
| chicken (*Gallus gallus*) | 2004 | International Chicken Genome Sequencing Consortium et al. 2004. Sequence and comparative analysis of the chicken genome provide unique perspectives on vertebrate evolution. Nature 432: 695-716 |
| dog (*Canis familiaris*) | 2005 | Lindblad-Toh et al. 2005. Genome sequence, comparative analysis and haplotype structure of the domestic dog. Nature 438: 803-19 |
| bee (*Apis mellifera*) | 2006 | Weinstock et al. 2006. Insights into social insects from the genome of the honeybee *Apis mellifera*. Nature 443: 931-949 |
| cat (*Felis catus*) | 2007 | Tamazian et al. 2014. Annotated features of domestic cat - *Felis catus* genome. Gigascience 3: 13. |
| cattle (*Bos taurus*) | 2009 | Bovine Genome Sequencing Consortium et al. 2009. The genome sequence of taurine cattle: a window to ruminant biology and evolution. Science 324: 522-8 |
| horse (*Equus caballus*) | 2009 | Wade et al. 2009. Genome sequence, comparative analysis, and population genetics of the domestic horse. Science 326: 865-7 |
| turkey (*Meleagris gallopavo*) | 2010 | Dalloul et al. 2010. Multi-platform next-generation sequencing of the domestic turkey (*Meleagris gallopavo*): genome assembly and analysis. PLoS Biology 8:e1000475 |
| salmon (*Salmo salar*) | 2010 | Davidson et al. 2010. Sequencing the genome of the Atlantic salmon (*Salmo salar*). Genome Biology 11: 403 |
| cod (*Gadus morhua*) | 2011 | Star et al. 2011. The genome sequence of Atlantic cod reveals a unique immune system. Nature 477: 207-10 |
| pig (*Sus scrofa*) | 2012 | Groenen et al. 2012. Analyses of pig genomes provide insight into porcine demography and evolution. Nature 491: 393-8 |
| goat (*Capra hircus*) | 2013 | Dong et al. 2013. Sequencing and automated whole-genome optical mapping of the genome of a domestic goat (*Capra hircus*). Nature Biotechnology 31: 135-41 |
| duck (*Anas platyrhynchos*) | 2013 | Huang et al. 2013. The duck genome and transcriptome provide insight into an avian influenza virus reservoir species. Nature Genetics 45: 776-83 |
| sole (*Cynoglossus semilaevis*) | 2014 | Chen et al. 2014. Whole-genome sequence of a flatfish provides insights into ZW sex chromosome evolution and adaptation to a benthic lifestyle. Nature Genetics 46: 252-260 |
| sheep (*Ovis aries*) | 2014 | Jiang et al. 2014. The sheep genome illuminates biology of the rumen and lipid metabolism. Science 344: 1168-1173 |
| rabbit (Oryctolagus cuniculus) | 2014 | Carneiro et al. 2014. Rabbit genome analysis reveals a polygenic basis for phenotypic change during domestication. Science 345: 1074-1079 |
| trout (*Oncorhynchus mykiss*) | 2014 | Berthelot et al. 2014. The rainbow trout genome provides novel insights into evolution after whole-genome duplication in vertebrates. Nature communications 5: 3657 |
| tilapia (*Oreochromis niloticus*) | 2014 | Brawand et al. 2014. The genomic substrate for adaptive radiation in African cichlid fish. Nature 513: 375-381 |

**Table S1: reference species and publications of livestock genomes.**
